# Interception of vertically approaching objects: temporal recruitment of the internal model of gravity and contribution of optical information

**DOI:** 10.3389/fphys.2023.1266332

**Published:** 2023-11-17

**Authors:** Sergio Delle Monache, Gianluca Paolocci, Francesco Scalici, Allegra Conti, Francesco Lacquaniti, Iole Indovina, Gianfranco Bosco

**Affiliations:** ^1^ Laboratory of Visuomotor Control and Gravitational Physiology, IRCCS Santa Lucia Foundation, Rome, Italy; ^2^ Department of Systems Medicine and Centre for Space BioMedicine, University of Rome Tor Vergata, Rome, Italy; ^3^ Laboratory of Neuromotor Physiology, IRCCS Santa Lucia Foundation, Rome, Italy; ^4^ Department of Biomedicine and Prevention, University of Rome Tor Vergata, Rome, Italy; ^5^ Brain Mapping Lab, Department of Biomedical and Dental Sciences and Morphofunctional Imaging, University of Messina, Messina, Italy

**Keywords:** manual interception timing, internal gravity representation, vestibular network, Bayesian regression, optical variables, looming, parallel processing, altered gravity

## Abstract

**Introduction:** Recent views posit that precise control of the interceptive timing can be achieved by combining on-line processing of visual information with predictions based on prior experience. Indeed, for interception of free-falling objects under gravity’s effects, experimental evidence shows that time-to-contact predictions can be derived from an internal gravity representation in the vestibular cortex. However, whether the internal gravity model is fully engaged at the target motion outset or reinforced by visual motion processing at later stages of motion is not yet clear. Moreover, there is no conclusive evidence about the relative contribution of internalized gravity and optical information in determining the time-to-contact estimates.

**Methods:** We sought to gain insight on this issue by asking 32 participants to intercept free falling objects approaching directly from above in virtual reality. Object motion had durations comprised between 800 and 1100 ms and it could be either congruent with gravity (1 g accelerated motion) or not (constant velocity or -1 g decelerated motion). We analyzed accuracy and precision of the interceptive responses, and fitted them to Bayesian regression models, which included predictors related to the recruitment of *a priori* gravity information at different times during the target motion, as well as based on available optical information.

**Results:** Consistent with the use of internalized gravity information, interception accuracy and precision were significantly higher with 1 g motion. Moreover, Bayesian regression indicated that interceptive responses were predicted very closely by assuming engagement of the gravity prior 450 ms after the motion onset, and that adding a predictor related to on-line processing of optical information improved only slightly the model predictive power.

**Discussion:** Thus, engagement of *a priori* gravity information depended critically on the processing of the first 450 ms of visual motion information, exerting a predominant influence on the interceptive timing, compared to continuously available optical information. Finally, these results may support a parallel processing scheme for the control of interceptive timing.

## Introduction

Catching or avoiding approaching objects are common daily actions that we can perform effortlessly. Over the years, a long standing debate has animated the literature about whether interceptive actions could be afforded entirely by optical variables derived directly from available visual signals, such as the ratio (τ) of the object’s image retinal size and its expansion rate originally proposed by Hoyle (1957) and Weinberger (1971), and reinvigorated by Lee (1976), or the distance between the approaching object and the observer (Collewijn, 1972; Carl and Gellman, 1987; Gellman and Carl, 1991; van Donkelaar et al., 1992; Port et al., 1997). In particular, Lee’s τ model, inspired by Gibson’s ecological approach (Lee, 1976; Gibson, 1979; Lee and Reddish, 1981), motivated much research in the manual interception field, receiving support from psychophysical work that applied and revisited the model to explain interceptive behavior in various experimental conditions (Bootsma and Oudejans, 1993; Peper et al., 1994; Rushton and Wann, 1999), as well as from neurophysiological evidence that neurons in the optic tectum of the pigeon may encode time-to-contact information in line with the τ model predictions (Sun and Frost, 1998).

Conversely, several experimental and theoretical works have challenged the assumptions of the τ model, by showing that it may not predict accurately time-to-contact information, depending on the acceleration, trajectory duration, texture, shape or size of the moving target (Lacquaniti and Maioli, 1989; Smeets et al., 1996; DeLucia et al., 2000; López-Moliner et al., 2007a; 2007b; Zago et al., 2009; Hosking and Crassini, 2010; 2011; Jacobs and Díaz, 2010; Lugtigheid and Welchman, 2011; López-Moliner and Keil, 2012). Furthermore, prior knowledge and cognitive factors, which are not taken into account by the τ model, can be also integrated into the processing of time-to-contact information, contributing greatly to interceptive outcome (Wann, 1996; Tresilian, 1999; López-Moliner and Bonnet, 2002; Baurès et al., 2018). This evidence, indeed, motivated variations of the τ model that included prior knowledge information, such as the KS model proposed by López-Moliner and others, which considered the known size of the object (López-Moliner et al., 2007b; López-Moliner and Keil, 2012).

Given that objects are constantly accelerated by Earth’s gravity and that the visual system is not very sensitive to accelerated motion (Werkhoven et al., 1992; Lisberger and Movshon, 1999; Priebe and Lisberger, 2002; Watamaniuk and Heinen, 2003), it has been hypothesized that the brain, through lifelong experience, may build an internal representation of gravity effects on the objects’ motion, which contributes to the predictive estimates of the time-to-contact with the approaching object (Lacquaniti and Maioli, 1989; Lacquaniti et al., 1993). A similar idea has been also formulated to account for the systematic downward offset in the direction of gravity, known as representational gravity, with which a moving target is perceived after the motion has vanished (Hubbard, 1995; De Sá Teixeira and Hecht, 2014; also Hubbard, 2020 for a review). This *a priori* expectation of natural 1g accelerated motion not only would account for the ability to successfully intercept objects accelerated by natural gravity, but it would also imply that, whenever contextual cues suggest gravity effects on the target motion, it could be used inappropriately regardless of the actual target kinematics (Zago et al., 2008; 2009; Lacquaniti et al., 2013; 2014; 2015; Jörges and López-Moliner, 2017; Delle Monache et al., 2021).

In line with this view, several studies have shown anticipation of the effects of gravity when intercepting targets moving downward at constant velocity along vertical path or parabolic trajectories (McIntyre et al., 2001; Zago et al., 2004; 2009; Senot et al., 2005; Bosco et al., 2012; Indovina et al., 2013a; La Scaleia et al., 2015; 2020; Russo et al., 2017). Moreover, neuroimaging studies have identified a potential neural correlate of the internal model of gravity in the activity of the multimodal vestibular network (Indovina et al., 2005; 2013b; 2015; Bosco et al., 2008; Miller et al., 2008; Maffei et al., 2010; Ferri et al., 2016; De Sá Teixeira et al., 2019). Encoding of *a priori* knowledge of gravity by the vestibular cortex might be related to the capacity of canonical cortical microcircuits to generate top-down predictive signals, which suppress the responsiveness of lower order areas to predictable stimuli, whereas unpredictable stimuli relayed by ascending feedforward excitatory signals would generate prediction errors (Friston, 2005; Bastos et al., 2012; Maffei et al., 2015; Bogacz, 2017). Further, transcranial magnetic stimulation studies have established causal links between the activity of one of the main hubs of the vestibular network in the temporoparietal junction (TPJ) and the timing of the interceptive response (Bosco et al., 2008; Delle Monache et al., 2017). Interestingly, in the study of Bosco et al. (2008), where TMS pulses were delivered either 100 or 300 ms after the target motion onset, causal effects were observed only immediately after the beginning of the object motion and not for the subsequent interval, underlining the primary role of predictive processes in the control of interceptive actions.

Owing to the strong experimental evidence that prior information contributes to the control of interceptive actions, a recent model proposed by López-Moliner and others for the interception of ballistic trajectories (the so-called GS model), assumes that time-to-contact estimates are derived from the combination of mechanisms based on the continuous update of optical variables with predictions based on prior knowledge of the object size and of the effects of gravity on the object motion (Gómez and López-Moliner, 2013; de la Malla and López-Moliner, 2015). By occluding either the earlier or the final portion of the ballistic trajectories, De La Malla and López-Moliner (2015) suggested further that predictive mechanisms may prevail at the beginning of the target trajectories, whereas on-line mechanisms may contribute more during the last portion of the trajectory, even though this parsing of information was not as clear-cut when the target was continuously visible (de la Malla and López-Moliner, 2015). Remarkably, by manipulating parametrically the value of the gravity acceleration, it was concluded that the gravity prior can be considered as a strong prior with a mean very close to 9.8 m/s^2^ and a standard deviation of 2.07 m/s^2^ (Jörges and López-Moliner, 2017; 2020).

However, these results, except for the aforementioned study by De La Malla and López-Moliner (2015), do not provide a clear indication on the relative contribution of internalized gravity and optical information in determining the time-to-contact estimates. Furthermore, although both the TMS findings by Bosco et al. (2008) and the psychophysical results by De La Malla and López-Moliner (2015) pointed out an early contribution of the gravity prior, it is not clear whether this prior could be engaged based exclusively on contextual cues (visual, vestibular, somatosensory) informing about the naturalness of the environment independently of the target motion, or it is actually reinforced by incoming target motion information. Finally, it should also be considered that, with the exception of few studies (Senot et al., 2005; de la Malla and López-Moliner, 2015; La Scaleia et al., 2019)–which manipulated the law of motion of a ball projected toward the observer (i.e., visual looming stimuli)–studies validating the idea of the internal model of gravity have generally used visual motion projected tangentially to the observer. In this respect, acquiring deeper knowledge on how the brain combines gravity prior information with incoming sensory signals by using more immersive virtual reality settings, may also impact on the understanding of the adaptive processes to altered gravity environments and on the identification of potential countermeasures to the space motion sickness (Arshad and Ferré, 2023; Bizzarri et al., 2023; Khalid et al., 2023).

Based on these premises, we sought to gain further insight by partially replicating the experimental setting of Senot et al. (2005), with, however, a more advanced virtual reality device, which allowed fuller 3D immersion in the visual environment. In brief, we simulated the fall of a ball from a tree toward a participant laying supine on the ground, and asked participants to intercept the passage of the ball through a ring positioned right above their head with a button press. Trials in which we simulated the effects of natural gravity (downward accelerated motion, 1g = 9.8 m/s^2^) were randomly intermixed with trials in which the motion of the object was not congruent with the effects of gravity, by falling at constant speed or decelerating (at -1g). We analyzed the interceptive responses with Bayesian regression models, which included predictors related to the recruitment of *a priori* gravity information at different times during the target motion, as well as to the use of available optical information. This modeling analysis pointed out that: 1) the timing of the interceptive responses was predominantly influenced by *a priori* gravity information; 2) early processing of visual motion information up to 450 ms after the target motion onset appeared crucial for engaging effectively the internal representation of gravity; 3) mechanisms based on continuous processing of optical variables contributed marginally to the interceptive timing.

## Methods

Thirty-two healthy individuals (18 women, 14 men, mean age 26.5 ± 7.2 SD), with normal or corrected-to-normal vision, volunteered to participate in the experiment. Sample size was determined based on prior studies involving roughly comparable conditions (Senot et al., 2005; La Scaleia et al., 2019). The experimental procedures adhered to the Declaration of Helsinki and were approved by the Ethics Committee of the IRCCS Santa Lucia Foundation (CE/PROG.906 20-01-21).

### Experimental set-up and task

Participants performed manual interception of visual looming stimuli in an immersive 3D virtual reality environment, created by using the Unity game engine (Unity Software, release 2019.4.4f1, CA, US). Visual stimuli were displayed through an Oculus Rift (Reality Labs, CA, US) controlled by a dedicated gaming laptop (Asus GL504G, Asustek, Taiwan), at a spatial resolution of 1,080 by 1,200 pixels per eye and with a refresh rate of 90 Hz. Participants wore the Oculus Rift while lying supine on an exercise mat. All participants reported stereoscopic view when tested before the experiment with a standard depth perception test, as well as when wearing the Oculus Rift and viewing the virtual reality scene.

**FIGURE 1:**
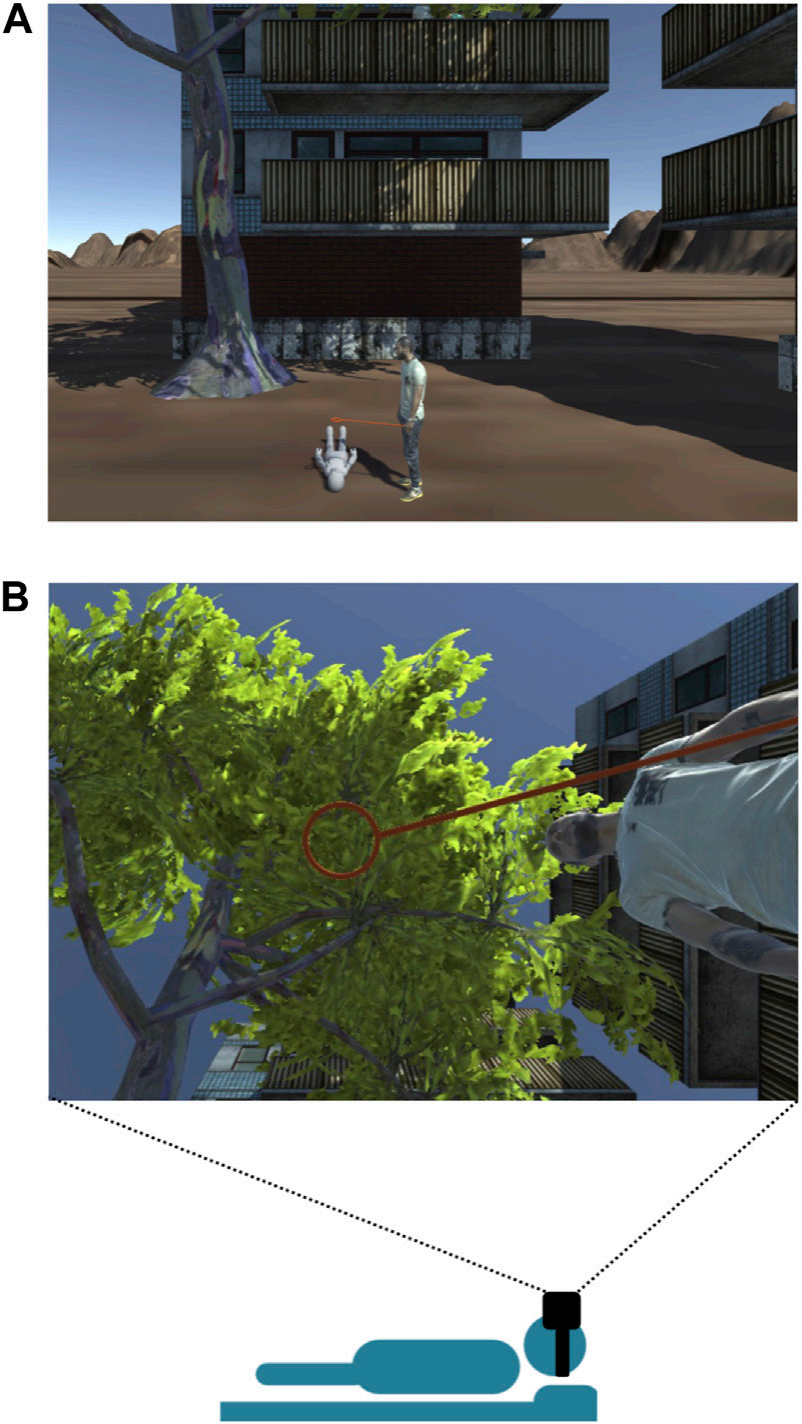
Visual scene and experimental task. **(A)**. The visual scene represented an avatar lying supine on the ground under a tree. Participants performed the interception task, while laying supine on a gym mat, like the avatar in the virtual environment. On the right side of the avatar, an adult male figure stood upright holding an orange stick with a ring at its end. **(B)**. Point of view of the scene from the avatar/participant perspective when lying supine and with the gaze directed at the center of the ring. On each trial, a red ball dropped vertically from the tree branches, approaching the participant’s head. Participants were instructed to press the right button of a computer mouse to intercept the ball as it passed through the ring, while maintaining the gaze fixed.

In the virtual reality scenario, the participant’s avatar laid supine on an exercise mat (∼5 cm tall) under a tree (see Figure 1). The avatar point of view in the virtual scenario was always congruent with the instantaneous position of the participant’s head. On the right side of the avatar, an adult male figure stood upright holding an orange stick with a 14 cm wide ring at its distal end. The human figure was displayed to help participants reaching a rough estimate of distances. The ring was held at 0.775 m above the ground, corresponding to about 0.5 m above the avatar’s head. Participants positioned their head below the virtual ring and directed their gaze straight up through the center of the ring, maintaining fixation on a designated point of the tree foliage placed at a virtual height of 9.72 m from the ground. At the beginning of each trial, a red ball, 7 cm diameter, roughly the size of a tennis ball, was dropped from the fixation point towards the avatar’s head, along the line of sight. Participants were instructed to intercept the ball as it passed through the ring, by pressing the right button of a customized computer mouse (Logitech, Switzerland). The ball disappeared from view 40 cm below the ring. Participants did not receive feedback on the outcome of the interceptive response.

**FIGURE 2:**
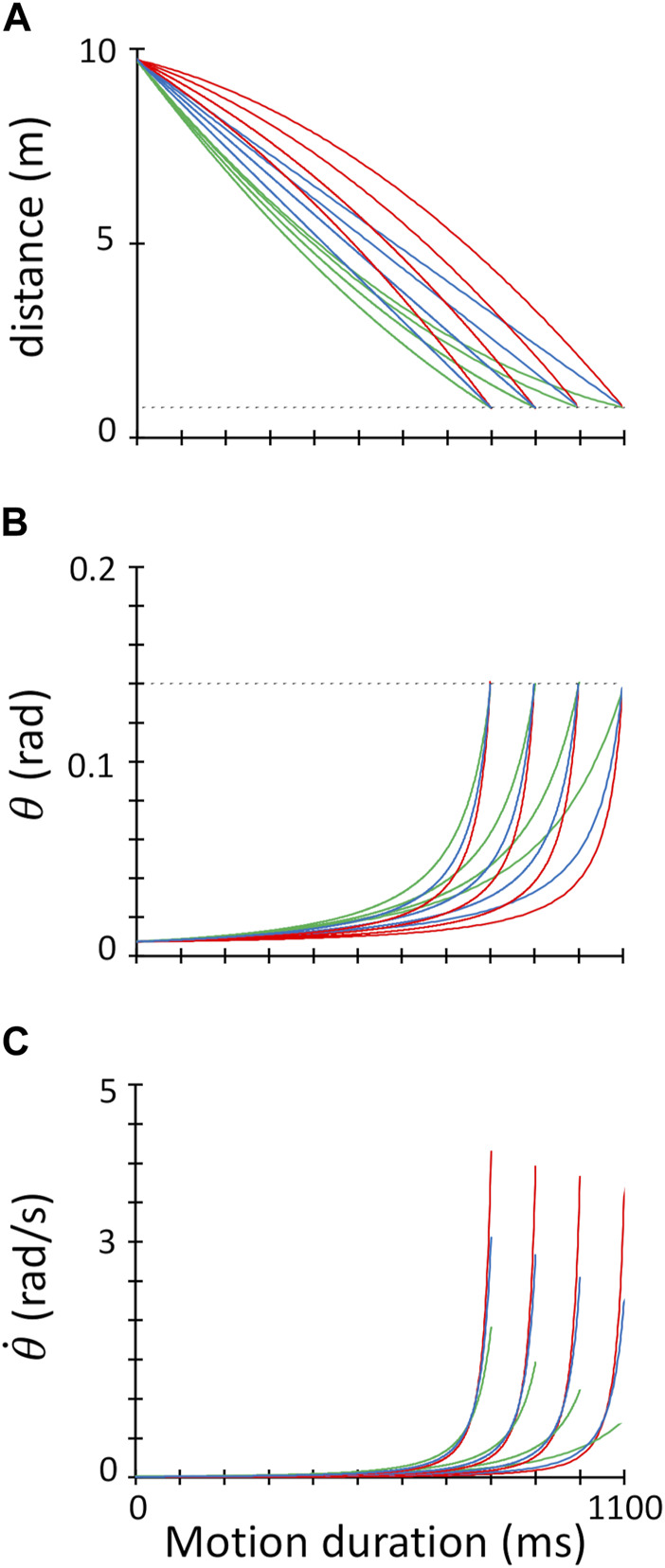
Target kinematics. **(A)**. The distance of the target from the interception point (in meters, considering a real size virtual scene) is plotted against the time from the target motion onset. Motion trajectories intersect the interception height (dashed grey line) at the four target motion durations (1,100 ms; 1,000 ms; 900 ms; 800 ms). Red, blue, and green traces refer to 1 g, 0 g and -1g trials, respectively. **(B)**. Time course of the retinal image size ( θ ) for the 12 experimental conditions. Same color coding as in **(A)**. **(C)**. Time course of the retinal image dilation rate ( $\dot{\theta }$ ) for the 12 target trajectories. Same color coding as in A-B.

The downward motion of the ball in each trial could follow one of three possible kinematic profiles: accelerated by gravity (1g: 9.8 m/s^2^, scaled to the virtual reality scene metrics), decelerated by the same amount (-1g) or constant velocity (0g). Thus, 1g ball motion simulated natural motion conditions under zero drag conditions (for an estimate of the effects of drag on a falling ball, see for instance Zago et al., 2008), while -1g and 0g represented altered gravity conditions. For each acceleration level (-1g | 0g | 1g), we set four possible motion durations (800, 900, 1,000 or 1,100 ms), by varying the ball initial velocity, so that the mean target velocity for each motion duration was equal across acceleration levels. This resulted in 12 different kinematic profiles (3 motion accelerations * 4 motion durations, Table 1 and Figure 2). For all trajectories, retinal image size changes were above the threshold for perceiving motion in depth starting from about the first 50 ms after the onset. During the experimental session, 20 repetitions of the 12 experimental conditions were presented in pseudorandom order for a total of 240 trials. Participants were familiarized with the task and with the virtual reality environment by performing, before the experimental session, 24 training trials consisting of 2 repetitions of each experimental condition distributed pseudo randomly.

**TABLE 1:** Kinematic parameters for the 12 experimental conditions. *a* accelerated 1g motion, *d* decelerated -1g motion, *c* constant velocity motion. V_0_ and V_t_ refer, respectively, to the initial and the terminal (at the interception point) velocity of the ball expressed in m*s^-1^.

Motion duration [s]	*a*	*d*	*c*
	V_0_/V_t_	V_0_/V_t_	V_0_/V_t_
1.1	2.7/13.5	13.5/2.7	8.1/8.1
1.0	4.0/13.8	13.8/4.0	8.9/8.9
0.9	5.5/14.3	14.3/5.5	9.9/9.9
0.8	7.3/15.1	15.1/7.3	11.2/11.2

The temporal distribution of the experimental trials and the acquisition of button-press responses were controlled by custom-made algorithms in MATLAB R2020b (Data acquisition toolbox, Mathworks, MA, US) running on a separate PC (master PC) from the one running the virtual reality environment. The two computers were interfaced through an NI USB-6218 I/O board (National Instrument, TX, US), controlled by the master PC and receiving TTL signals at the beginning of the trial from the laptop running the virtual reality environment through a USB serial interface (TTL-232RG, FTDI, UK), as well as the TTL signals related to the participants’ button press responses (sampling rate: 10 kHz).

### Data processing

For each trial, we computed the timing error (TE) as the difference between the button-press response time (RT) and the time at which the ball crossed the ring (the ideal RT). Thus, negative, and positive TE values denoted anticipated and delayed responses, respectively. For each participant, we first discarded the trials in which TE values exceeded two standard deviations from the mean TE for a given experimental condition (mean 10.03 ± 2.83 discarded trials for each participant; total number of trials discarded across participants: 321/7680, 4.2%). Then, we computed the mean TE values and standard deviations (std TE) for each experimental condition as measures of interception accuracy and precision, respectively.

The datasets obtained by pooling mean and std TEs across experimental conditions and participants were submitted to full factorial repeated measure ANOVAs with motion acceleration (MA: 1g | 0g | 1g) and motion duration (MD: 800 | 900 | 1,000 | 1,100 ms) as “within subjects” factors. For the sake of the interpretation of the model results, it is worth pointing out that the motion duration is inversely related to the mean target velocity. The significance level was set at *p* < 0.05, Greenhouse-Geisser corrected.

### Bayesian linear mixed regression models

In order to investigate the temporal course of the recruitment of the gravity internal model and the relative contribution of optical information to the participants’ interceptive timing, we used inferential statistics, submitting a dataset of 384 mean RT values (32 participants * 12 experimental conditions) to Bayesian linear mixed regression models (brms package in R; for details see Bürkner, 2017). In brief, Bayesian regressions estimated the values of the dependent variable–i.e., the observed RT values–from a family of posterior probabilities (likelihood estimates) obtained from multiple draws (n = 4,000) of the data and by applying model priors to the regression coefficients’ distributions.

First, we addressed the issue of the time-course of the internal model of gravity recruitment, by including in the Bayesian regression model only “*gravity*” predictors, which were based on the idea that participants could engage the *a priori* assumption that targets were all accelerated by gravity either from the very beginning of the target motion or after a certain interval of time, implying early visual motion processing. Thus, we created thirteen different *gravity* predictors by considering time intervals spaced every 50 ms, from target motion onset (t = 0) to the first 600 ms and then by computing for the target motion conditions with altered gravity (-1g, 0g) the corresponding estimated times of arrival of the targets as if they moved at 1g from that time point on. Note, in fact, that for 1g motion conditions the assumption of gravity effects on the target motion of the *gravity* predictors was congruent with the actual law of motion of the target. Thus, regardless of the time point at which the gravity prior was engaged in the different *gravity* predictors, they all returned correct ball free falling duration estimates. Instead, for -1g and 0g targets, assumption of gravity effects would result in increasingly shorter temporal estimates of the ball free falling duration going from 0g to -1g targets, and, ultimately, in increasingly earlier interceptive responses (see Table 2).

**TABLE 2:** Bayesian linear mixed regression model predictors. A subset of the series of *gravity* predictors generated by assuming that the internal model of gravity was engaged at given time points during the target trajectory is represented by the columns G-ons to G-600. The subset includes predictors assuming time points spaced 100 ms apart from the motion onset to 600 ms after. The optical predictor estimated the time of arrival of the ball at the interception point from optical variables and known object size (KS model, see text for details). Predictors’ values are expressed in milliseconds.

MA	MD	G-ons	G-100	G-200	G-300	G-400	G-500	G-600	Optical
**-1g**	1,100	551	608	665	721	777	832	887	1,063
	1,000	541	597	651	705	758	810	859	971
	900	528	580	632	682	731	777	820	879
	800	508	557	605	650	692	731	765	784
**0g**	1,100	755	802	846	889	929	967	1,001	1,100
	1,000	717	760	800	839	875	907	936	1,000
	900	674	713	749	783	814	841	864	900
	800	627	661	693	721	746	768	784	801
**1g**	1,100	1,100	1,100	1,100	1,100	1,100	1,100	1,100	1,112
	1,000	1,000	1,000	1,000	1,000	1,000	1,000	1,000	1,012
	900	900	900	900	900	900	900	900	911
	800	800	800	800	800	800	800	800	811

The effects of each of the thirteen *gravity* predictors were tested by fitting the observed RT values with thirteen separate Bayesian linear mixed regression models, which included fixed and random effects of the *n*th *gravity* predictor and a random effect variable (*subjects*) accounting for variable intercepts across participants. We included both fixed and random effects for the *gravity* predictor, to discriminate between the effects for each experimental condition (fixed effects) and those in the whole population (random effects). The general formulation of the Bayesian regression model in R-language syntax was the following:

$$\text{mean RT}∼{gravity}_{\left(n\right)}+\left({gravity}_{\left(n\right)}\left.+1\right|subjects\right)$$

where *gravity*_*(n)*_ represented the *n* ^th^ *gravity* predictor, and the terms between parenthesis were related to the random effects. The model prior used for the coefficient of the fixed effect of the *gravity* predictor was a normal distribution with μ = 0 and σ = 1, whereas for the random effects, we used the brms default Student t-distribution priors (Bürkner, 2017). The goodness of fit of each of the thirteen Bayesian regression models was evaluated by the Bayesian *R* ^2^, which is related to the overall dataset variance explained by the model, and by the expected error variance σ^2^ (Gelman et al., 2019).

Next, we assessed the additional contribution of optical information by including another fixed effect predictor (“*optical”*) to the Bayesian regression model best fitting the observed RT with one of the *gravity* predictors. The *optical* predictor was based on the idea that time-to-contact information was also derived from available optical information, such as the retinal image dilation rate ( $\dot{\theta }$ ). In essence, it represented estimates of the time of target arrival at the interception point obtained by applying to each target motion condition the KS model proposed by López-Moliner et al. (2007b); López-Moliner and Keil (2012):

$$TTC\approx \frac{1}{\sqrt{{\dot{\theta }}_{th}}}\sqrt{\frac{s}{v}},\text{from which }{\dot{\theta }}_{th}=\frac{s}{vT_{ca}^{2}}$$

where TTC was the time-to-contact with the visual target (in s), $T_{ca}$ was the time interval necessary to execute the button press action, s the size of the ball in meters, ${\dot{\theta }}_{th}$ the retinal image dilation rate threshold for triggering the interceptive action (in rad/s), v the physical target velocity (m/s). This latter variable was derived from the retinal image size ( θ ), its dilation rate and the physical size of the object according to the relationship:

$$v=\frac{s\dot{\theta }}{\theta^{2}}$$

For each target motion condition, we obtained the retinal image expansion rate threshold ${\dot{\theta }}_{th}$ , by considering a $T_{ca}=150\;\text{ms}$ and the mean target velocity ( v ) during 100 ms preceding $T_{ca}$ (i.e., between 150 and 250 ms before the target arrival at the interception point). By applying these parameters to the KS model, the resulting *optical* predictor estimated shorter free falling times for -1g targets (producing earlier interceptive responses), slightly longer free falling times for 1g targets (producing delayed interceptive responses), and accurate timing for 0g conditions (see Table 2). Thus, as shown by Table 2, the ball free falling duration estimates based on either the *gravity* or *optical* predictors were different for all types of motion, but particularly divergent with respect to -1g and 0g motion. For the coefficient of the fixed effect of the *optical* predictor we specified the same model prior as the *gravity* predictor, namely, a normal distribution with μ = 0 and σ = 1.

Finally, we applied the Leave-one-out (LOO) cross-validation procedure (Vehtari et al., 2017) to compare the predictive performance of the Bayesian linear mixed regression models either with or without the *optical* predictor and determine whether its inclusion improved significantly the model predictions. An expected log pointwise predictive density (ELPD) difference between the two models greater than 4 and exceeding the Standard Error was considered as statistically significant (Sivula et al., 2022).

## Results

We evaluated the participants’ interceptive performance with visual looming targets either compatible (1g motion) or not (-1g, 0g motion) with natural gravity effects, by measuring interception accuracy (mean TE) and precision (std TE), as well as by fitting the individual response times to Bayesian linear mixed regression models.

### Interception accuracy

**FIGURE 3:**
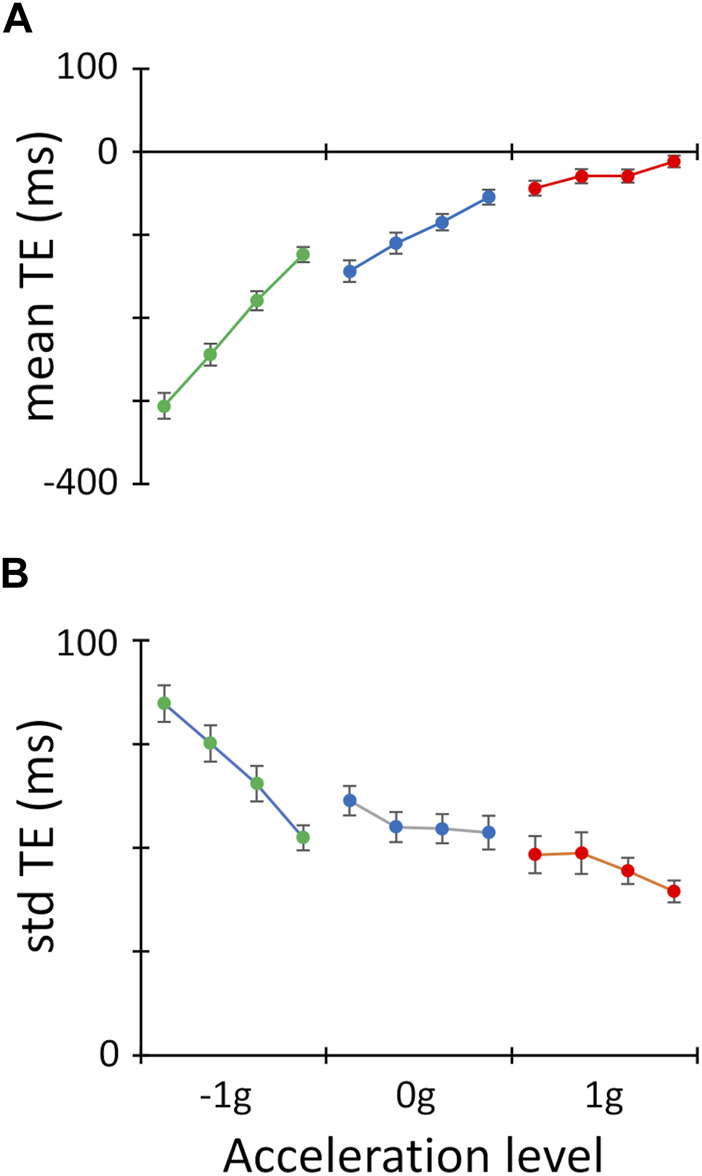
Interception accuracy and precision. **(A)**. Mean TE values ±SEM (interception accuracy) computed across participants for each experimental conditions are plotted with respect to the twelve experimental conditions. Data points are grouped for target acceleration (red: 1 g; blue: 0 g; green: 1g) and, within each target acceleration level, they are ordered for target duration (from left to right 1,100 → 800 ms). **(B)**. Same layout as A for std TE values ±SEM (interception precision).

The interception accuracy was influenced strongly by the targets’ acceleration (main effect of MA in Table 3), being higher with 1g motion (red symbols in Figure 3A), and progressively lower with 0g (blue symbols) and -1g motion (green symbols), due to the increasingly earlier interceptive timing in response to these latter two types of motion. Ball motion duration was also a significant factor affecting the interception accuracy (main effect of MD in Table 3), as progressively earlier responses were observed with longer motion durations. This monotonic scaling of the interceptive timing with the motion duration was, however, more pronounced for -1g and 0g motion than for 1g motion, accounting for the statistically significantly two-way interaction MA*MD.

**TABLE 3:** Results of the repeated measures ANOVAs comparing mean TE (A) and TE standard deviation values (B) pooled across experimental conditions and participants (significant factors at *p* < 0.050 Greenhouse-Geisser corrected, in bold).

Factor	dof		F		p
MA	2		931.177		**< 0.001**
MD	3		295.777		**< 0.001**
MA*MD	6		127.726		**< 0.001**

Factor	dof		F		p
MA	2		85.841		**< 0.001**
MD	3		26.821		**< 0.001**
MA*MD	6		6.889		**< 0.001**

### Interception precision

Significant effects of ball motion acceleration and duration were observed also for the std TE, an inverse measure of interception precision (Figure 3B; Table 3). The interceptive response variability increased progressively with accelerated, constant velocity and decelerated targets, as well as from the shortest (800 ms) to the longest motion durations (1,100 ms). This monotonic trend with respect to the motion duration was, again, more pronounced for -1g and 0g trials than for 1g trials (two-way interaction MA*MD). Overall, the better interception accuracy and precision observed for 1g compared to 0g and -1g trials, suggests that visual looming objects that are congruent with gravity effects engaged the internal representation of gravity, thereby providing a performance advantage with this type of motion.

### Bayesian linear mixed regression models

The results of the ANOVAs on the interception accuracy and precision could not provide insight on the temporal course with which the internal representation of gravity was engaged, or on the extent to which on-line mechanisms driven by the available optical information were also at play. To address these issues, we adopted inferential statistics and submitted the observed RT values to Bayesian linear mixed regression models, which included predictors based on the assumption of gravity effects on the target motion and on optical variables.

**FIGURE 4:**
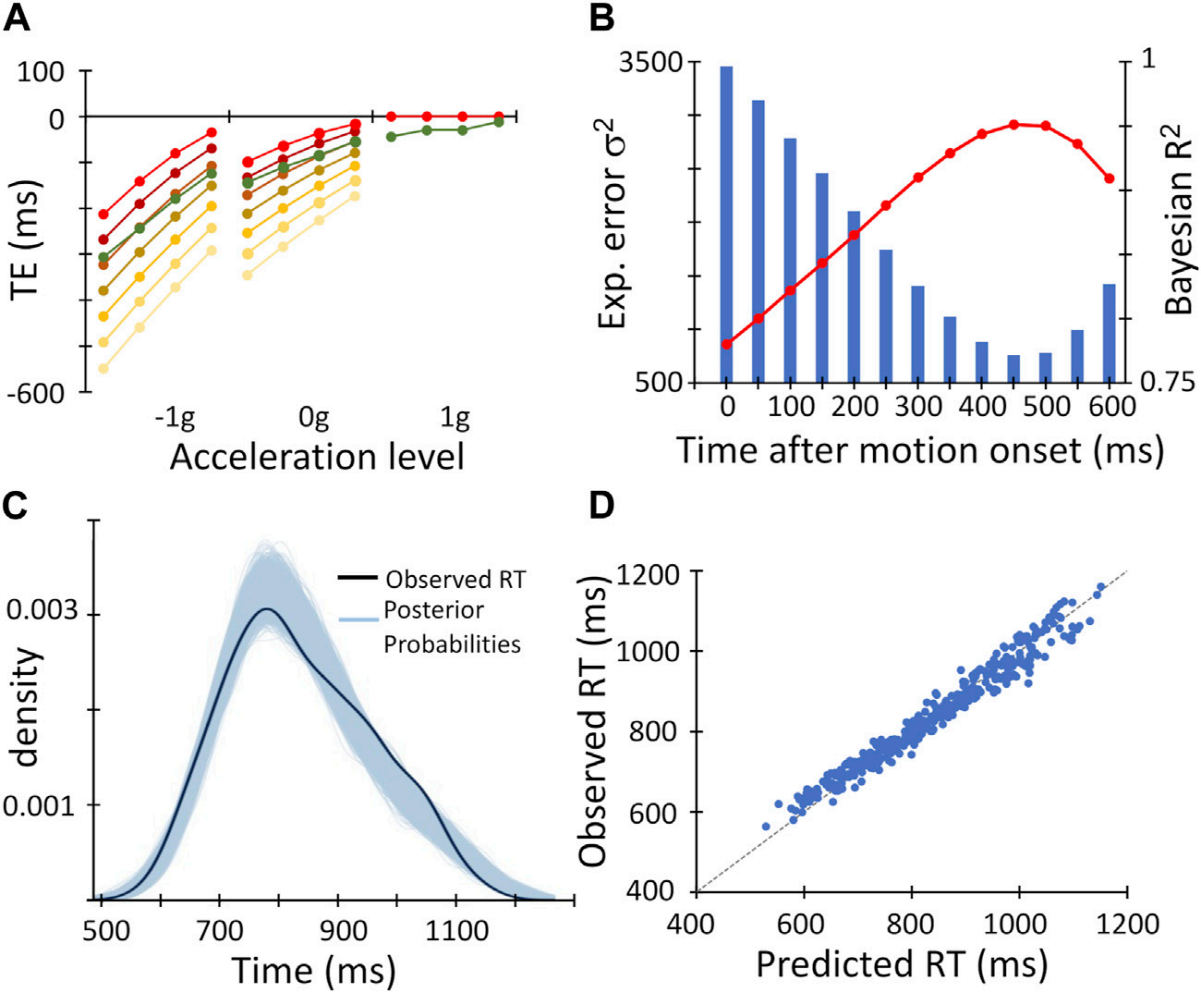
Results of the time-course analysis of Bayesian regression models with the *gravity* predictors. **(A)** *gravity* predictors (yellow-to-red symbols), converted to the corresponding timing errors, are overlaid on the observed mean timing errors (dark green symbols, same datapoints as in Figure 3A). Note that the *gravity* predictor curves coincide perfectly in the 1g domain, since all of them assume correct interception (TE = 0). Therefore, for the sake of clarity, only the red symbols are shown. **(B)**. The expected error variance σ^2^ (blue bars) and the Bayesian *R* ^2^ (red symbols and line) resulting from the model fits with the thirteen *gravity* predictors are plotted against the time from the target motion onset for the recruitment of the internal model of gravity applied to each *gravity* predictor. The primary and secondary *Y*-axes refer to the expected error variance and the Bayesian *R* ^2^, respectively. **(C)**. The observed distribution of mean Response Time (RT) values across experimental conditions and participants (black curve) is overlaid on the family of 4,000 posterior probabilities resulting from the Bayesian regression (sky-blue curves). **(D)**. The observed mean Response Time (RT) values across experimental conditions and participants are plotted against the values predicted by the Bayesian linear mixed regression model. The unitary slope line is represented by the light grey dashed line.

We found a clear temporal course in the recruitment of the gravity internal representation by fitting participants’ RT values with a series of thirteen Bayesian regression models, which included different *gravity* predictors related to the engagement of the gravity *a priori* at specific time points from the target motion onset (*t = 0*) to 600 ms thereafter. As illustrated by Figure 4A with a subset of the *gravity* predictors (yellow to red symbols) plotted as timing errors, the TE values associated with the *gravity* predictors appeared increasingly closer to the observed mean TEs (green symbols) when the time elapsed before engaging the gravity *a priori* was comprised between 400 and 500 ms. This qualitative observation was substantiated quantitatively by the series of Bayesian *R* ^2^ and expected error variances resulting from the model fits (Figure 4B). The lowest expected error variance and the highest Bayesian *R* ^2^ occurred, in fact, with the *gravity* predictor assuming an interval of 450 ms. This Bayesian regression model accounted for about 95% of variance in the RT dataset (Bayesian *R* ^2^ = 0.951; see Table 4 for complete model results), and predicted remarkably well the observed RT values, as their distribution fell well within the family of 4,000 posterior probability distributions resulting from the Bayesian regression (Figure 4C), and the datapoints in the plot of observed vs. predicted values were distributed uniformly around the unitary slope line (Figure 4D).

**TABLE 4:** Results of Bayesian linear mixed models. A. Model including only the *gravity* predictor assuming that the gravity *a priori* was engaged 450 ms after the beginning of the target motion. B. Model including both the gravity and the optical predictor.

*Fixed Effects*					
	Estimate	Est.Error	Rhat	Bulk_ESS	Tail_ESS
**Intercept**	−8.8	16.32	1.01	687	1,510
**gravity**	0.99	0.01	1	5,112	2,914
***Random Effects***					
**sd(Intercept)**	68.08	13.44	1.01	805	1,080
**sd(gravity)**	0.02	0.01	1.01	575	1,019
**cor(Intercept, gravity)**	−0.5	0.49	1	2,181	2,112
***Family Specific Parameters***					
**sigma**	27.57	1.07	1	3,693	2,616

*Fixed Effects*					
	Estimate	Est.Error	Rhat	Bulk_ESS	Tail_ESS
**Intercept**	−30.22	17.47	1	902	1814
**gravity**	0.93	0.02	1	3,437	3,105
**optical**	0.07	0.02	1	3,513	3,191
***Random Effects***					
**sd(Intercept)**	67.19	12.57	1.01	919	1,583
**sd(gravity)**	0.02	0.01	1.01	449	889
**cor(Intercept, gravity)**	−0.49	0.48	1	2,160	2,364
***Family Specific Parameters***					
**sigma**	27.1	1.03	1	4,982	3,012

**FIGURE 5:**
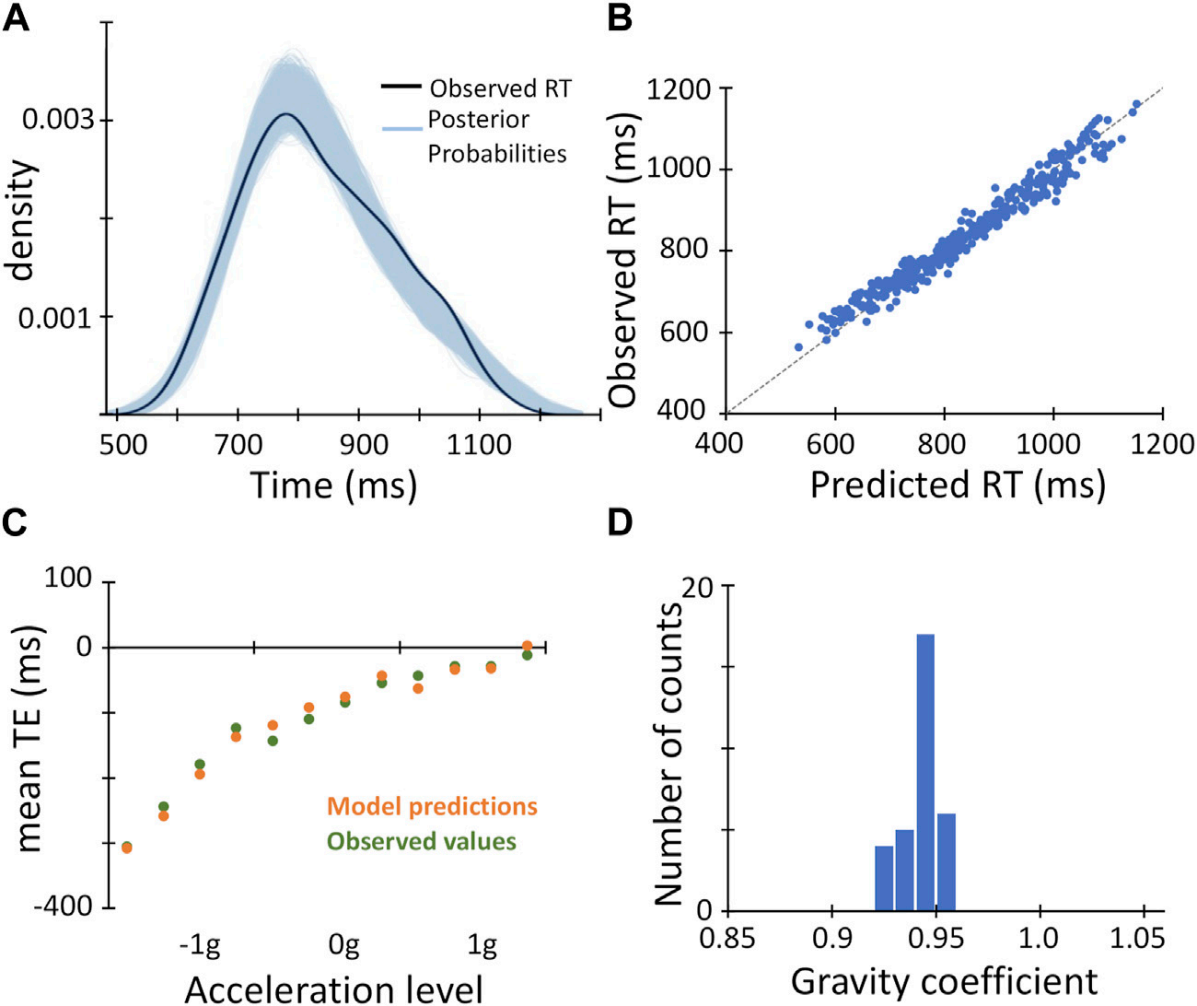
Results of Bayesian linear mixed regression model including *gravity* and *optical* predictors. **(A)**. The observed distribution of mean Response Time (RT) values across experimental conditions and participants (black curve) is overlaid on the family of 4,000 posterior probabilities resulting from the Bayesian linear mixed regression model (sky-blue curves). **(B)**. The observed mean Response Time (RT) values across experimental conditions and participants are plotted against the values predicted by the Bayesian linear mixed regression model. As in Figure 4D, the light grey dashed line represents the unitary slope line. **(C)**. The observed mean TE values (dark green symbols, same datapoints as in Figure 3A) and the mean TE values predicted by the Bayesian linear mixed regression model are plotted against the experimental conditions, grouped for target acceleration, and ordered for target motion duration (from left to right 1,100 → 800 ms). **(D)**. Distribution of the *gravity* predictor regression coefficient estimated by the Bayesian regression model across participants.

To evaluate whether also available optical information contributed to the interceptive timing, we included in this Bayesian linear mixed regression model another predictor (*optical*)*,* based on the assumption that time-to-contact information could be derived from optical variables and known object size (López-Moliner et al., 2007b; Jörges and López-Moliner, 2017). This latter Bayesian regression model fitted very closely the observed RT values, explaining, however, only slightly higher fraction of the variance than the model including just the *gravity* predictor (Bayesian *R* ^2^ = 0.953; Table 4; Figures 5A–C). Furthermore, the regression coefficient of the fixed effect of the *gravity* predictor was very close to unity and distributed with a rather small variance across participants (Table 4; Figure 5D). Indeed, by comparing the two models directly with the Leave-one-out cross-validation procedure, we found slightly higher expected predictive accuracy for the model that also included the *optical* predictor (ELPD difference = 5.9 ± 4.4 SE), indicating that its addition improved the model predictive power to a rather small, though significant, degree.

## Discussion

This study investigated the relative contribution of *a priori* gravity information and of continuously available optical information to the interception of looming visual objects in vertical free-fall, approaching the observer from above. To this end, we presented visual looming stimuli in virtual reality and manipulated the kinematics to be either congruent or not with the effects of natural gravity. We evaluated accuracy and precision of participants’ interceptive responses and fitted them to Bayesian linear mixed regression models, including predictors related to the recruitment of *a priori* gravity information at different times during the target motion, as well as to the use of available optical information.

The first main finding was that object motion congruent with the effects of gravity was intercepted with greater accuracy and precision than constant velocity and decelerated motion, suggesting that the participants could estimate time-to-contact information predictively based on internalized gravity information (Lacquaniti and Maioli, 1989; McIntyre et al., 2001; Bosco et al., 2012). This result goes along with earlier psychophysical and neuroimaging evidence suggesting that an internal representation of the gravity acceleration, derived from multimodal sensory information (i.e., visual, vestibular and somatosensory signals), and residing in the vestibular network is engaged by visual stimuli congruent with the gravity effects during both perceptual and motor tasks (Indovina et al., 2005; 2015; Bosco et al., 2008; Maffei et al., 2010; 2015; Moscatelli and Lacquaniti, 2011; De Sá Teixeira et al., 2019; Delle Monache et al., 2019). Indeed, this internal representation can be retrieved effectively not only in real life situations, but also by abstract representations of visual objects’ motion rendered against a uniform background or within a pictorial natural setting presented on two-dimensional flat-screen video projections or with visually immersive virtual reality devices (Miller et al., 2008; Delle Monache et al., 2015; 2019; 2021; Hubbard, 2020).

Further analysis of the interceptive responses with Bayesian linear mixed regression models revealed some remarkable aspects concerning how the internal gravity representation might be engaged and its relative contribution to the interceptive timing, given that also mechanisms based on the available optical information might be at play (Zago et al., 2009; Gómez and López-Moliner, 2013). First, a clear time course for the engagement of the gravity prior emerged by fitting the interceptive responses to different predictors related to the assumption that the internal model of gravity could be engaged at different time-points along the target trajectory. The best fit was found with a predictor related to a time-point 450 ms after the beginning of the target motion and this Bayesian regression model could predict very closely the observed behavior, explaining over 95% of the variance in the interceptive responses. This evidence may be analogous to the observation that the size of the downward displacement in the direction of gravity of the memorized location of a vanishing moving target (representational gravity) increased with the length of the retention interval, from 200 ms reaching asymptote at about 800 ms, indicating a time course in the recruitment of the internal model of gravity (De Sá Teixeira and Hecht, 2014; De Sá Teixeira, 2016). Moreover, this time course could be altered by vestibular stimulation, compatible with the involvement of vestibular brain areas (De Sá Teixeira et al., 2017). Similarly, our result may provide insight to the understanding of how the internal representation of gravity might be engaged during manual interceptions, as it implies strongly that early processing of visual motion signals up to 450 ms after the target motion onset played a crucial role.

In fact, in an earlier TMS study we reported that dual pulse stimulation of TPJ - a core region of the vestibular network repository of the internal gravity representation - produced significant effects on the interceptive timing only when delivered at 100 ms after the target motion onset, thereby disrupting cortical activity for the following 200 ms (Indovina et al., 2005; Bosco et al., 2008). However, this early time window of TPJ inactivation could be compatible with two possible scenarios: 1) that *a priori* gravity information was recruited in preparation of the upcoming interceptive action before any significant processing of visual motion information by the vestibular cortex could occur, as the effect of the first TMS pulse delivered 100 ms after the beginning of the target motion may potentially imply; 2) alternatively, since the effects of the dual pulse TMS extended for further 200 ms, visual motion information processed by TPJ within the first 300 ms of the trial could have played a role. The fact that, in the present study, retinal image size changes were above the threshold for perceiving motion in depth starting from about the first 50 ms after the onset (Regan and Beverley, 1979; Wann et al., 2011) and that a longer visual processing window of 450 ms emerged from the time-course analysis, indicate definitely that visual motion information, combined with other multisensory cues informing about the overall congruence of the virtual reality scene to a realistic setting, was indeed responsible for engaging effectively activity in the vestibular network, putting the *a priori* gravity cues into play (Gallagher et al., 2020; Delle Monache et al., 2021).

The second striking aspect emerging from the Bayesian regression analysis was that inclusion of a predictor related to the available optical information improved only slightly the predictive power of the model, suggesting a predominant contribution of predictive mechanisms based on the assumption of gravity effects for the control of the interceptive timing, at least under the present experimental conditions. This finding was somewhat unexpected given the simple, directly approaching, visual looming stimulus we used. Indeed, there appears to be some consensus in the literature that, at least for objects approaching at constant speed, on-line mechanisms based on optical variables, as modeled by the *optical* predictor in our Bayesian regression, may suffice under many conditions (Gómez and López-Moliner, 2013).

On the other hand, the only earlier studies to our knowledge adopting parametric manipulation of the gravity effects in the motion of directly approaching looming stimuli comparable to that used in the present study, are those carried out by Senot et al. (2005; 2012). Noteworthy, some expected analogies, as well as some remarkable differences, did become evident by comparing the results of our study with that by Senot and others using vertical rectilinear trajectories directly approaching the observer (Senot et al., 2005). The earlier study found higher success rates with constant velocity targets and comparable fits of the interceptive responses with the predictions of the τ model and of a model that considered the use of the internal model of gravity starting at a given time threshold (λ) at which the interceptive action was triggered. Because the distribution of the interceptive responses to 0g motion deviated systematically from the predictions of the τ model and the interceptive timing was influenced also by the target motion direction relative to gravity, the authors concluded that *a priori* gravity information contributed significantly to the interceptive timing.

Here, we observed a similar systematic relationship between the response timing to 0g targets and the target motion duration (i.e., mean target velocity), which does not seem to be entirely compatible with an interception model based on optical variables, such as the τ model. Moreover, the distribution of the interceptive timing in response to 0g and to -1g targets was clearly more in line with the estimates of the *gravity* than the *optical* predictor. Indeed, unlike the study by Senot and others, we found much better interception accuracy and precision with 1g compared to 0g motion, and better fit of the observed response times with predictions based on the use of internalized gravity information.

In effect, this apparent incongruency could be related to different approaches undertaken by the two studies in terms of the experimental design and the modeling analysis. With respect to the experimental design, the present study employed only downward motion, while the earlier study presented the same ball kinematics in both vertical directions, either falling from above or ascending from below. Moreover, even though both studies involved button press responses to intercept the moving targets, in the study by Senot et al., participants had to account for an additional delay of about 57 ms introduced by the fact that the button press triggered the motion of a virtual racquet, with which the ball was intercepted. Another difference concerned the availability of online visual feedback of the interceptive outcome, provided in the experiment of Senot et al., but not in ours. In sum, one possibility we might consider in order to explain the interceptive performance differences between the two studies is that the higher number of experimental conditions, the potential higher task difficulty related to the additional response delay, and the presence of visual feedback could have made the experiment conducted by Senot and others more prone to a central tendency effect, which would be compatible with the better performance observed when participants intercepted 0g motion.

Regarding the modeling analysis, the λ model used by Senot et al. to fit the observed response times posits that the assumption that all targets were accelerated by gravity could be applied once a fixed time threshold (λ) before enacting the interceptive response was reached, implying that internalized gravity information might be embedded in the time-to-contact countdown process. With the Bayesian regression models, we took a rather different approach, by considering that the *a priori* assumption of gravity effects on the target motion could be applied at any time-point from the target motion onset up to 600 ms thereafter, thus independently of a time threshold set by a countdown process. This may also imply that the internal representation of gravity in the vestibular cortex might bias, with variable strength depending on the situation, an independent countdown process based on continuously available visual information, which was exemplified by the *optical* predictor in our Bayesian regression model. Specifically, from the modeling results it appeared that, at least for our experimental situation, internalized gravity information exerted a very strong bias on the countdown process, given the marginal contribution of the *optical* predictor to the predictive power of the Bayesian regression model.

From a neurophysiological standpoint, the findings of our modeling analysis, in effect, may support the parallel processing scheme proposed by Delle Monache et al. (2017) for the control of interceptive actions. By interpreting the current results within this functional framework, we might hypothesize that information determining the participants’ time-to-contact estimates would result from processing along two separate pathways. One pathway might involve visual motion areas, such as hMT/V5+, which would feed timing information based on optical variables to downstream posterior parietal areas (Battelli et al., 2007; Bosco et al., 2008; Bueti et al., 2010; Dessing et al., 2013; Salvioni et al., 2013; Delle Monache et al., 2017; Baurès et al., 2021). In the other pathway, visual signals about the initial target motion, combined with other sensory signals informing about the physical properties of the environment, would engage the internal representation of gravity in the multimodal areas of the vestibular network (Indovina et al., 2005; 2013a; 2013b; Zago et al., 2008; 2009; Lacquaniti et al., 2013; Bosco et al., 2015; Maffei et al., 2015; Delle Monache et al., 2021). The two pathways may, finally, converge in premotor/motor cortical areas, contributing independently to the build-up activity of cortical motor areas related to motor timing (Merchant et al., 2004a; 2004b; 2009; 2011; Merchant and Georgopoulos, 2006; Merchant and Averbeck, 2017; Merchant and Bartolo, 2018). In this respect, we suggest that the build-up of activity itself could reflect the time-to contact countdown based on optical information carried by the first pathway, whereas gravity prior information through the second pathway could exert a level bias on the activity in build-up neurons, thus affecting the threshold for triggering the interceptive response.

## Conclusion

In sum, two main conclusions may be drawn from the findings reported here: 1) *a priori* gravity information exerted a strong predictive bias over countdown mechanisms based on available optical information for the control of the interceptive timing in response to vertically looming objects; 2) the internal representation of gravity was engaged with a time-course, which implied processing of early visual motion information up to 450 ms after the beginning of the target motion. Finally, insights provided by these results on how the brain may combine sensory signals and *a priori* information about the physical properties of the environment to drive motor behavior in simulated altered gravity conditions may be also relevant to a better comprehension of the mechanisms underlying motor adaptation to altered gravity environments.

## Data Availability

The raw data supporting the conclusion of this article will be made available by the authors, without undue reservation.
